# Impact of lifestyle factors on adult-onset asthma in genetically high-risk individuals

**DOI:** 10.7189/jogh.15.04147

**Published:** 2025-06-06

**Authors:** Shin Young Kwon, Ji Eun Lim, Hae-Un Jung, Eun Ju Baek, Hyein Jung, Ji-One Kang, Tae-Bum Kim, Bermseok Oh

**Affiliations:** 1 Department of Biomedical Science, Graduate School, Kyung Hee University, Seoul, Republic of Korea; 2 Department of Biochemistry and Molecular Biology, School of Medicine, Kyung Hee University, Seoul, Republic of Korea; 3 Fortuna Helix, Seoul, Republic of Korea; 4 Department of Allergy and Clinical Immunology, Asan Medical Centre, University of Ulsan College of Medicine, Seoul, Republic of Korea; * Joint first authorship.; † Equal contribution.; ‡ Joint senior authorship.

## Abstract

**Background:**

Asthma is a heterogeneous disease influenced by genetic and environmental factors. Identifying high-risk individuals for developing asthma is a major goal of primary prevention. Recent studies have reported that genetic factors can predict high-risk groups for various diseases. However, the understanding of genetically high-risk individuals for adult-onset asthma, which could be potential targets for preventive interventions, remains limited. Using a polygenic risk score (PRS), we identified a group of individuals at a genetically high risk for adult-onset asthma and examined their characteristics, as well as the degree of risk reduction through lifestyle factors.

**Methods:**

Using the asthma PRS provided by the UK Biobank, we identified a genetically high-risk group among unrelated White British individuals, including those with adult-onset asthma and a control group. We analysed the association between asthma-related lifestyle factors and adult-onset asthma in the high-risk group. Additionally, we conducted Kaplan-Meier analysis to investigate the cumulative incidence of adult-onset asthma by age of onset according to genetic risk and lifestyle status.

**Results:**

The high-risk group for adult-onset asthma had a 2.26 times higher risk of developing asthma compared to the average-risk group. Within the high-risk group, lifestyle factors, such as obesity-related traits, stress, insomnia, and snoring, were significantly associated with adult-onset asthma. Among individuals in the high-risk group, adhering to a favourable lifestyle can reduce the lifetime risk of adult-onset asthma by up to 40% compared to those with an unfavourable lifestyle.

**Conclusions:**

Based on this study, we suggest that the PRS can identify individuals at high risk of developing adult-onset asthma. Furthermore, our findings indicate that individuals with a genetically high risk may reduce their disease risk through appropriate lifestyle modifications, emphasising their potential to benefit significantly from primary prevention strategies. However, as this study is cross-sectional and assumes that lifestyle factors have remained unchanged, further prospective studies are required to validate these findings.

Asthma is a common chronic disease affecting over 300 million people worldwide. It constitutes a significant burden on individual health, the global economy, and health care systems [1]. Despite decades of public health efforts through primary prevention guidelines, the worldwide incidence of asthma is increasing [2–4], indicating the pressing need for effective interventions to prevent asthma [5].

Asthma is a heterogeneous disease with a combination of genetic and environmental factors [6]. The age of onset is an important factor in distinguishing the subtypes of asthma [7]. Adult-onset asthma is more heterogeneous, often severe, and frequently associated with the loss of lung function [8]. Several twin studies have suggested that the genetic contribution to the predisposition to adult-onset asthma is lower than that to childhood asthma, with heritability estimates ranging from 57–73% in adults and 82% in children [9,10]. This suggests that non-genetic factors may play a greater role in adult-onset asthma than in childhood asthma. Many of these factors may contribute to adult-onset asthma through cumulative exposure over many years rather than short-term exposure. The risk factors related to a modifiable lifestyle may be important for the primary prevention of adult-onset asthma. Lifestyle-related risk factors for asthma include physical activity, smoking, mental health, obesity, sleep disorders, and socioeconomic status [11–16].

Identifying individuals or groups at high risk of developing asthma is a major goal of primary prevention [17]. Genetic factors are stable and inherent and can be used to identify and guide interventions in individuals at high risk of developing adult-onset asthma from an early age. Recently, approaches using the polygenic risk score (PRS) have been actively studied to identify high-risk groups for diseases [18,19]. The PRS aggregates numerous common variants weighted by the impact of each allele on disease risk. The PRS can be used as an indicator to identify individuals with higher genetic susceptibility to diseases [20]. Khera et al. screened a genetically high-risk group for common diseases, such as coronary artery disease, type two diabetes, and inflammatory bowel disease, using the PRS and observed higher prevalence rates than in genetically normal groups [21]. Although efforts have been made to predict the risk of asthma using the PRS [22], there have been insufficient studies on the genetic risk of adult-onset asthma. Accordingly, our understanding of genetically high-risk individuals for adult-onset asthma, who could be potential targets for preventive interventions, and the effectiveness of primary prevention efforts in these individuals, remains limited.

The primary aim of this study was to understand genetically high-risk individuals for adult-onset asthma. For this purpose, we identified a genetically high-risk group for adult-onset asthma and investigated their characteristics and degree of risk reduction through lifestyle modification. We conducted this analysis using data from the UK Biobank (UKB), including asthma PRS and demographic, clinical, and medical history data of nearly 500 000 participants.

## METHODS

### Study population and design

The UKB is a large population-based cohort of approximately 500 000 individuals aged 40–69 years across the UK, established to investigate the interrelationships among the environment, lifestyle, and genetics [23]. In this study, we used data collected from participants who attended the initial assessment centre visit (2006–10). For sample quality control, we applied the Neale laboratory inclusion criteria to our initial samples as follows [24]: 1) participants used to compute the genetic principal components (PCs) (field identifier (ID) 22020), 2) participants determined to be of British ancestry based on PCs (field ID 22009), and 3) ‘White-British,’ ‘Irish,’ and ‘White’ participants based on the self-reported ethnic background (field ID 22009). Additionally, we included participants with non-missing standard PRS for asthma (field ID 26210) and age at recruitment between 40–69 years (field ID 21022).

**Figure 1:**
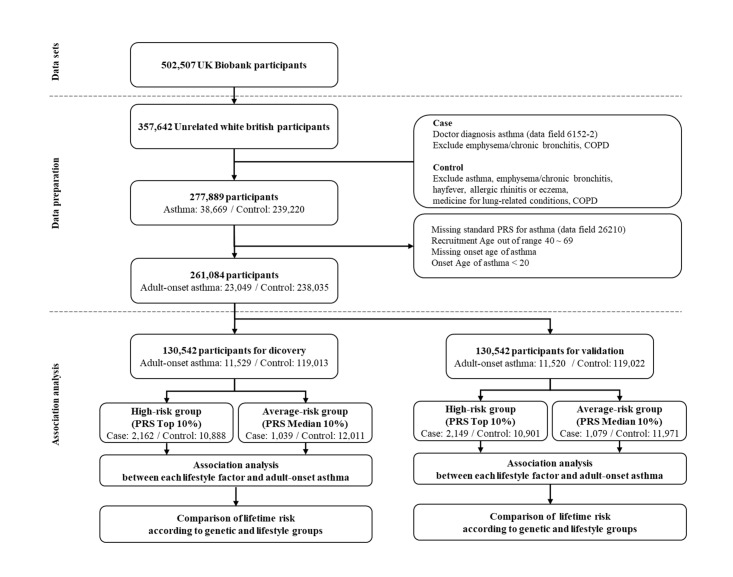
Study design flowchart. COPD – chronic obstructive pulmonary disease, PRS – polygenic risk score.

From the 357 642 unrelated White British individuals in the UKB, we selected 23 049 with adult-onset asthma (onset age, 20–69 years) and 238 035 controls (**Figure 1**).

Individuals with adult-onset asthma were defined as individuals who self-reported having physician-diagnosed asthma (field ID 6152). We excluded participants with emphysema, chronic bronchitis (field ID 6152), and chronic obstructive pulmonary disease (COPD) (field ID 22130, 41202). Additionally, we excluded participants who had an asthma onset before the age of 20 years or had missing information on the age of asthma onset (field ID 3786). We defined controls as individuals excluding those who self-reported diagnoses of emphysema, chronic bronchitis, hay fever, allergic rhinitis, or eczema; those with self-reported diagnoses or International Classification of Diseases, 10th revision codes for COPD and asthma; and those on medication for lung-related conditions (field ID 20003) [25]. For further analysis, we randomly divided 261 084 participants into two equal-sized groups – discovery and validation data sets.

### Standard polygenic risk score for asthma from UKB

The UKB released the PRS for 53 diseases and quantitative traits through project number 9659 [26]. We used the standard PRS for asthma (field ID 26210) provided by the UKB to select a genetically high-risk group for adult-onset asthma. We generated the standard PRS for asthma using a Bayesian approach applied to meta-analysed summary statistics from external genome-wide association studies (GWAS) data (electronic medical records and genomics (emerge) network cohort data), and we calculated it for all individuals in the UKB.

To select the genetically high- and average-risk groups for adult-onset asthma, we divided study participants into percentiles based on their PRS, and asthma prevalence within each group was investigated. We defined the top 10% and median (MD) 10% of the PRS as the high- and average-risk groups, respectively (Figure S1 in the **Online Supplementary Document**).

### Lifestyle risk factors

To investigate the lifestyle factors influencing individuals with a high genetic risk for adult-onset asthma, we selected 41 candidate lifestyle factors reported to be associated with asthma based on a comprehensive literature review (Table S1 in the **Online Supplementary Document**). These candidate lifestyle factors were obtained from a list of various environmental factors related to asthma used in previous study analyses, and only lifestyle-related factors were selected [27]. To incorporate a broader range of lifestyle factors, an extensive literature review was conducted, resulting in the inclusion of additional categories such as local environment, sun exposure, and sleep [16,28–32]. Subsequently, we included only factors for which more than 70% of individuals in our study population, who are at high genetic risk for adult-onset asthma, had available data. Consequently, we considered a total of 41 candidate lifestyle factors. These lifestyle factors consisted of the following eight categories: diet (nine factors), obesity (10 factors), stress (one factor), sociodemographic factors (one factor), physical activity (11 factors), local environment (two factors), sun exposure (four factors), and sleep (three factors). For the quality control of factors, we used the interquartile range (IQR) method (3 × IQR rule) to remove outliers for each factor with continuous values.

After investigating the association between adult-onset asthma and each lifestyle factor in the genetically high-risk group, we constructed a comprehensive lifestyle risk score using the following significantly associated factors: body mass index (BMI), neuroticism score, insomnia, and snoring. We first classified the lifestyle status of each factor as favourable, intermediate, or unfavourable for each individual (Table S2 in the **Online Supplementary Document**). Lifestyle risk scores were calculated as the number of factors with unfavourable lifestyle status.

Additionally, we constructed a weighted lifestyle risk score for the four lifestyle risk factors to confirm the robustness of the study results. The formula for the weighted lifestyle risk score was as follows:

Weighted lifestyle risk score = (*β*_BMI_ × status of BMI + *β*_neuroticism score_ × status of neuroticism score + *β*_insomnia_ × status of insomnia + *β*_snoring_ × status of snoring) × (4/(*β*_BMI_ + *β*_neuroticism score_ + *β*_insomnia_ + *β*_snoring_)) [33].

We calculated the weight *(β*) for each lifestyle factor using a logistic regression model adjusted for age and sex in the discovery data set (Table S3 in the **Online Supplementary Document**). We used the resulting weights to calculate the weighted lifestyle risk scores for individuals in the validation data set. Individuals were then categorised into three groups according to the weighted lifestyle risk score as follows: favourable (lowest quintile), intermediate (quintiles two to four), and unfavourable (highest quintile) lifestyle.

### Statistical analysis

We used a logistic regression model adjusted for covariates, including sex, age, genotyping array, and 10 PCs, for the association analysis. We used *t* tests and χ^2^ tests to compare the characteristics between controls and individuals with adult-onset asthma. We determined statistical significance after adjusting *P*-values using the Bonferroni correction for multiple testing. Further, we used Kaplan-Meier analysis to compare the cumulative incidence of adult-onset asthma outcomes among the genetic and lifestyle risk groups. As data from these pooled study cohorts were cross-sectional, cumulative incidence plots were constructed for individuals with adult-onset asthma using the onset age of asthma (field ID 3786). Thus, the follow-up time was from age 20 years to the age at diagnosis of adult-onset asthma or age at the time of the initial assessment visit (2006–10), whichever occurred first. From these data, we estimated the lifetime risk for adult-onset asthma for the UKB population [34,35].

We calculated the sample size testing for this study using G*Power, version 3.1.9.7 (Heinrich Heine University Düsseldorf, Düsseldorf, Germany) [36]. For the logistic regression analysis, the parameters were set with a probability of asthma in the average-risk group of 0.08, an alpha error probability of 0.001, a power of 95%, and an odds ratio of 2.3, representing the genetic risk of adult-onset asthma in the high-risk group compared to the average-risk group. We set the *R^2^* value for other covariates (age and sex) to 0.04 due to their low association with PRS groups. The distribution of the predictor variable (PRS group) and the proportion of the high-risk group were set to binomial (high-risk group and average-risk group) and 0.5, respectively.

We performed association analysis, *t* test, χ^2^ test, Kaplan-Meier analysis, odds ratio (OR) plotting, and cumulative incidence plotting using *R*, version 4.1.0 (R Core Team, Vienna, Austria). We used the ‘ggplot2’ package to draw the OR plot, and ‘survival’ and ‘survminer’ packages to obtain the cumulative incidences and draw the plots, respectively.

## RESULTS

### Characteristics of individuals with adult-onset asthma and controls in the UKB

The 261 084 individuals included 23 049 individuals with adult-onset asthma (63.16% female) and 238 035 controls (52.46% female) (Table S4 in the **Online Supplementary Document**). Individuals with adult-onset asthma had higher eosinophil count and percentage, as well as BMI. Additionally, the prevalence of obesity and respiratory symptoms, such as wheeze or whistling, cough on most days, and bring up sputum on most days, was higher in the asthma group than in the control group. The forced expiratory volume in one second (FEV1), forced vital capacity (FVC) ratio (FEV1/FVC ratio), and FEV1 percentage predicted were lower in the asthma group than in the control group.

### Association of asthma PRS with adult-onset asthma

**Figure 2:**
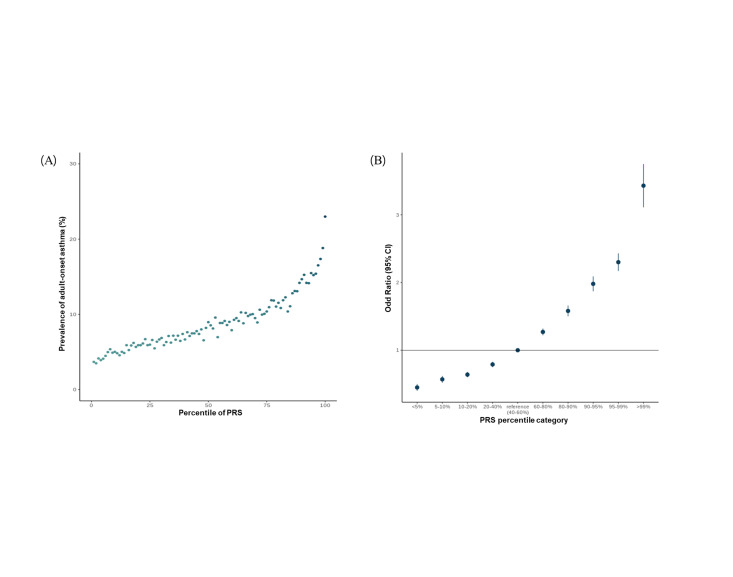
Risk for adult-onset asthma according to polygenic risk score (PRS). **Panel A.** Prevalence of asthma in each percentile group. **Panel B.** The odds ratios were calculated with a logistic model adjusted for age and sex by comparing each PRS group with a reference group (PRS percentile 40–60%). CI – confidence interval, PRS – polygenic risk score.

We investigated the risk of adult-onset asthma by stratifying 261 084 individuals into percentiles based on their PRS. We used the PRS (field ID 26210) provided by the UKB, constructed using external GWAS summary statistics that did not contain the UKB samples. The prevalence of adult-onset asthma gradually increased as the PRS increased (**Figure 2**, Panel A). Similar to the trend of increasing asthma prevalence, ORs gradually increased with increasing PRS. Compared to the 40–60% PRS group as a reference, the ORs ranged from 0.45 (95% confidence interval (CI) = 0.40–0.50; *P* < 3.2E–50) for the lowest PRS group (<5%) to 3.43 (95% CI = 3.11–3.78; *P* < 2.2E–137) for the highest PRS group (>99%) (**Figure 2**, Panel B; Table S5 in the **Online Supplementary Document**). In particular, the risk of adult-onset asthma increased sharply at the upper end of the PRS percentile. Therefore, we selected the top 10 percentiles of the PRS as the genetically high-risk group and investigated their characteristics, focusing on lifestyle risk factors.

### Association of lifestyle factors with adult-onset asthma in the genetically high-risk group

To investigate the characteristics of the genetically high-risk group, we randomly divided the 261 084 participants into two equal-sized groups – discovery and validation data sets. For each data set, 130 542 individuals were stratified according to PRS percentile. We arbitrarily defined the top 10 percentile (PRS>90) as the ‘high-risk’ group (n = 13 050) and the MD 10 percentile (PRS = 46–55) as the ‘average-risk’ group (n = 13 050) for reference (Figure S1 in the **Online Supplementary Document**). We computed the required sample size for this study using G*Power, version 3.1.9.7 [36]. With an alpha error probability of 0.001 and a power of 95%, the minimum sample size was calculated to be 1473 participants. Therefore, our sample size was adequate for detecting meaningful effects.

The genetically high-risk group had a higher risk of adult-onset asthma (OR = 2.26; 95% CI = 2.13–2.38) compared to the average-risk group (**Table 1**). We also found significant differences between the case groups in the high- and average-risk groups. Compared to the average-risk group, individuals with adult-onset asthma in the high-risk group showed: 1) an earlier onset age by 1.7 years, 2) 7.2% higher rates of hay fever, allergic rhinitis, or eczema, and 3) higher levels of eosinophil count and percentage by 0.04 (109 cells/L) and 0.6%, respectively. All these differences were statistically significant, with a *P* < 3.6E–03 (0.05/14) based on the Bonferroni correction. We also found significant differences between the control groups in the high- and average-risk groups. Compared to controls in the average-risk group, controls in the high-risk group had: 1) higher levels of eosinophil count and percentage by 0.02 (109 cells/L) and 0.28%, respectively, 2) higher levels of BMI and obesity prevalence by 0.25 (kg/m2) and 1.73%, respectively, 3) a 2.69% higher rate of wheeze or whistling, and 4) a 0.48% lower FEV1/FVC ratio and 1.75% lower FEV1 percentage predicted. All these differences were statistically significant, with a *P* < 4.2E–03 (0.05/12). These findings were also observed in the validation data set (Table S6 in the **Online Supplementary Document**).

**Table 1:** Basic characteristics of the high-risk and average-risk groups in the discovery data set*

	High-risk group		Average-risk group	
**Characteristics**	**Control (n = 10 888)**	**Case (n = 2162)**	**Control (n = 12 011)**	**Case (n = 1039)**
Age in years, x̄ (SD)†	57.27 (7.84)	57.01 (7.95)	56.95 (7.93)	56.67 (7.86)
Onset age in years, x̄ (SD)‡	NA	40.62 (12.27)	NA	42.33 (12.09)
Onset age in years				
*20–29*	NA	456 (21.09)	NA	171 (16.46)
*30–39*	NA	503 (23.27)	NA	235 (22.62)
*40–49*	NA	588 (27.20)	NA	280 (26.95)
*50–59*	NA	459 (21.23)	NA	263 (25.31)
*60–69*	NA	156 (7.22)	NA	90 (8.66)
Female§	5642 (51.82)	1327 (61.38)	6389 (53.19)	683 (65.74)
Male§	5246 (48.18)	835 (38.62)	5622 (46.81)	356 (34.26)
FEV1/FVC ratio†§	76.62 (8.43)	74.51 (9.82)	77.1 (8.37)	74.69 (9.66)
FEV1% predicted, x̄ (SD)†§	95.45 (15.86)	87.82 (17.94)	97.19 (15.75)	88.31 (17.74)
Eosinophil percentage, x̄ (SD)†‡§	2.61 (1.51)	3.33 (1.82)	2.33 (1.41)	2.73 (1.61)
Eosinophil count (10^9^ cells/L), x̄ (SD)†‡§	0.17 (0.1)	0.22 (0.12)	0.15 (0.10)	0.18 (0.11)
BMI (kg/m^2^), x̄ (SD)†§	27.47 (4.56)	28.35 (5.1)	27.22 (4.44)	28.2 (5.09)
Obesity†§	2669 (24.69)	679 (31.68)	2740 (22.96)	329 (31.85)
Hayfever, allergic rhinitis or eczema‡§	NA	963 (44.54)	NA	388 (37.34)
Wheeze or whistling†§	1618 (15.15)	1496 (70.04)	1472 (12.46)	681 (66.83)
Cough on most days§	283 (11.01)	136 (26.51)	323 (10.91)	81 (31.03)
Bring up sputum on most days§	171 (6.65)	90 (17.54)	185 (6.25)	30 (11.49)

BMI – body mass index, FEV1 – forced expiratory volume in one second, FVC – forced vital capacity, NA – not applicable, SD – standard deviation, x̄ – mean

*Presented as n (%) unless specified otherwise. Obesity BMI≥30 kg/m2. Eosinophil percentage is the proportion of eosinophils in the leukocytes. The mean or percentage was calculated for individuals with non-missing values.

†*P* < 4.2E–03 (0.05/12), comparison of controls between the high-risk and average-risk groups.

‡*P* < 3.6E–03 (0.05/14), comparison of cases between the high-risk and average-risk groups.

§*P* < 4.2E–03(0.05/12), comparison of case and control in each group.

We performed logistic regression analysis to investigate the association between lifestyle risk factors and adult-onset asthma in the high-risk group (Table S7 in the **Online Supplementary Document**). We adjusted the model for covariates, including age, sex, and PCs. Owing to the differences in the distribution of PRS between the high- and average-risk groups, we also included PRS as a covariate in the model. Among the 41 modifiable lifestyle factors previously reported to be associated with asthma, the following lifestyle factors in the high-risk group were significantly associated with an increased risk of adult-onset asthma (threshold *P*  = 1.2E–03 (0.05/41)): 1) higher measurements of factors related to obesity (BMI, weight, waist circumference, hip circumference, trunk fat mass, trunk fat percentage, whole-body fat mass, and body fat percentage), 2) higher neuroticism score, 3) higher levels of insomnia, and 4) presence of snoring (**Table 2**). These lifestyle factors were also significantly associated with adult-onset asthma in the average-risk group (*P* < 4.5E–03 (0.05/11)) (Table S8 in the **Online Supplementary Document**). All factors showed slightly larger effect sizes in the average-risk group than in the high-risk group; however, this difference was not statistically significant. Furthermore, we validated these findings using the validation data set with a threshold *P* = 2.3E–03 (0.05/22) (Table S9 in the **Online Supplementary Document**). Snoring showed a marginal association with the average-risk group with a *P* = 0.026.

**Table 2:** Factors significantly associated with adult-onset asthma in the high-risk group

	High-risk group (n = 13 050)		
**Category and lifestyle factor**	***β***	**SE**	*P*-value*
Obesity			
*BMI*	0.0422	0.0049	6.0E–18
*Weight*	0.0107	0.0016	4.7E–11
*Waist circumference*	0.0195	0.0019	6.8E–25
*Hip circumference*	0.0173	0.0026	1.5E–11
*Trunk fat mass*	0.0376	0.0046	3.2E–16
*Trunk fat percentage*	0.0274	0.0034	1.4E–15
*Whole body fat mass*	0.0213	0.0026	9.9E–17
*Body fat percentage*	0.0342	0.0039	1.0E–18
Stress			
*Neuroticism score*	0.0433	0.0082	1.5E–07
Sleep			
*Insomnia*	0.2189	0.0340	1.2E–10
*Snoring*	0.2068	0.0516	6.1E–05

BMI – body mass index, SE – standard error

*P < 9.8E–04 (0.05/41).

### Lifetime risk according to lifestyle in the genetically high-risk group

We investigated whether lifestyle modifications can reduce the lifetime risk of adult-onset asthma. Among the 11 lifestyle factors associated with adult-onset asthma in the high-risk group, eight obesity-related factors, including BMI, weight, waist and hip circumference, trunk fat mass and percentage, whole-body fat mass, and body fat percentage, were correlated with each other. Therefore, BMI was chosen as the representative factor for obesity-related traits because it is commonly used to define overweight and obesity in clinical practice and epidemiological studies in both sexes [37]. Further analyses were conducted using the following four lifestyle factors: BMI, neuroticism score, insomnia, and snoring. We categorised three lifestyle factors, BMI, neuroticism score, and insomnia, as follows: favourable, intermediate, or unfavourable. Snoring was categorised into two statuses: favourable and unfavourable (Table S2 in the **Online Supplementary Document**). We then calculated the lifestyle risk score for each individual by summing the number of unfavourable lifestyle factors among the four lifestyle factors. Based on the lifestyle risk score, we categorised overall lifestyle status as unfavourable (having at least three unfavourable lifestyle factors), intermediate (having one or two unfavourable lifestyle factors), or favourable (having no unfavourable lifestyle factors) (Table S10 in the **Online Supplementary Document**) [38,39].

We performed an association analysis of genetic and lifestyle groups with adult-onset asthma. We calculated ORs with 95% CI for three lifestyle categories (unfavourable, intermediate, and favourable) in each genetic group (high- and average-risk groups) compared to the reference group (average-risk with favourable lifestyle) (**Table 3**). The highest risk of asthma was observed among high-risk with an unfavourable lifestyle group (OR = 4.64; 95% CI = 3.75–5.76). The risk of adult-onset asthma increased gradually from favourable to unfavourable lifestyle status in both genetic risk groups. Asthma risks were similar between the average-risk with an unfavourable lifestyle group (OR = 2.47; 95% CI = 1.94–3.14) and the high-risk with a favourable lifestyle group (OR = 2.58; 95% CI = 2.12–3.15). Similar findings were also observed in the validation data set.

**Table 3:** Risk of adult-onset asthma according to genetic and lifestyle risk groups

	Discovery data set		Validation data set	
**Group**	**OR (95% CI)**	***P*-value**	**OR (95% CI)**	***P*-value**
High genetic risk				
*Unfavourable lifestyle*	4.64 (3.75–5.76)	6.15E–45	4.02 (3.26–4.97)	3.81E–38
*Intermediate lifestyle*	3.23 (2.72–3.86)	2.58E–39	3.05 (2.58–3.62)	1.34E–37
*Favourable lifestyle*	2.58 (2.12–3.15)	7.03E–21	2.47 (2.04–3.00)	3.47E–20
Average genetic risk				
*Unfavourable lifestyle*	2.47 (1.94–3.14)	2.42E–13	2.39 (1.88–3.03)	6.03E–13
*Intermediate lifestyle*	1.42 (1.18–1.72)	2.06E–04	1.34 (1.12–1.62)	1.35E–03
*Favourable lifestyle*	ref		ref	

CI – confidence interval, OR – odds ratio, ref – reference

**Figure 3:**
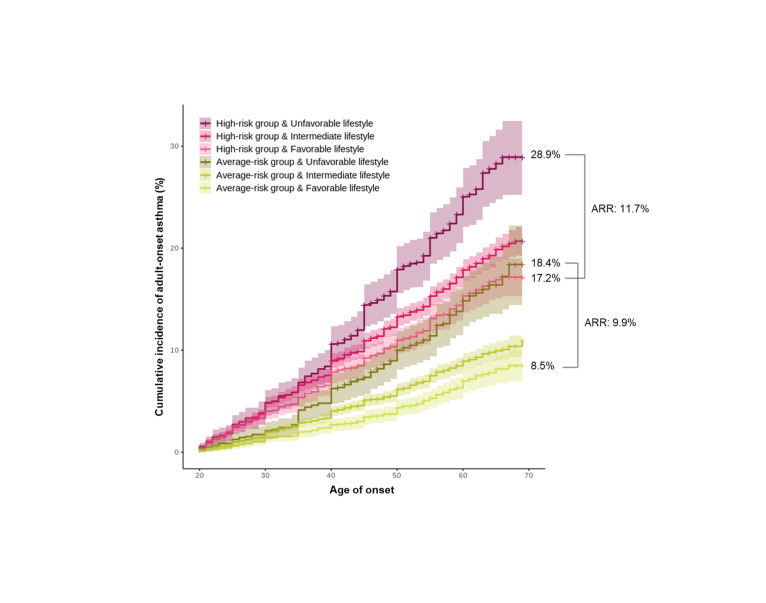
Cumulative incidence rates of adult-onset asthma according to genetic and lifestyle risk. Favourable (score 0), intermediate (score 1–2), and unfavourable (score 3–4) lifestyles were defined based on the lifestyle risk scores. Absolute risk reduction (ARR) is the difference between the lifetime risk of individuals with a favourable lifestyle and those with an unfavourable lifestyle within each genetic risk group.

We assessed the effects of genetic and lifestyle risk on the lifetime risk of asthma, analysing the cumulative incidence rates for each group according to genetic and lifestyle risks (**Figure 3**). In the high-risk group, the estimated cumulative lifetime risk was 28.9% in the unfavourable lifestyle group and 17.2% in the favourable lifestyle group. The absolute risk reduction (ARR) due to lifestyle was 11.7%. In the average-risk group, the estimated lifetime cumulative incidence risk was 18.4% for the unfavourable lifestyle group and 8.5% for the favourable lifestyle group (ARR = 9.9). This trend was replicated in the validation data set, with an ARR of 11.4% in the high-risk group and 11.0% in the average-risk group (Figure S2 in the **Online Supplementary Document**). The rate of risk reduction owing to lifestyle modifications was similar in both the high- and average-risk groups.

In addition, we conducted a sensitivity analysis using a weighted lifestyle risk score and compared the estimated lifetime cumulative incidence risk in each genetic and lifestyle risk group. We obtained the *β* coefficients of four lifestyle factors from the discovery data set (Table S3 in the **Online Supplementary Document**) and applied them as weights to individuals in the validation data set. We divided participants into three groups according to their weighted lifestyle risk scores: favourable (lowest quintile), intermediate (quintiles two to four), and unfavourable (highest quintile). In the validation data set, there was no significant difference in the ARR between the high-risk (ARR = 7.7) and average-risk groups (ARR = 8.3) (Figure S3 in the **Online Supplementary Document**). We also separately compared the estimated lifetime cumulative incidence risk for each lifestyle factor, including BMI, neuroticism score, insomnia, and snoring. In both the discovery and validation data sets, we observed a reduction in the lifetime risk when modifying each lifestyle factor (Figure S4–5 in the **Online Supplementary Document**). However, the modifying effect on snoring was minimal.

## DISCUSSION

We defined individuals at a genetically high risk of adult-onset asthma based on the asthma PRS and investigated the effectiveness of lifestyle modifications. We observed that eight obesity-related traits (BMI, weight, waist circumference, hip circumference, trunk fat mass, trunk fat percentage, whole body fat mass, and body fat percentage), stress (neuroticism score), insomnia, and snoring were lifestyle risk factors for adult-onset asthma in the genetically high-risk group. Modification of these lifestyle factors reduced the lifetime risk of adult-onset asthma in the genetically high-risk group (28.9% to 17.2%; ARR = 11.7) to an extent similar to that in the genetically average-risk group (18.4% to 8.5%; ARR = 9.9).

The genetically high-risk group had more than twice the risk of developing adult-onset asthma compared to the genetically average-risk group (OR = 2.26; 95% CI = 2.13–2.38), and showed significant differences in asthma characteristics. Individuals with asthma in the high-risk group had higher blood eosinophil counts than those in the average-risk group and were more likely to have allergies and atopy. In addition, non-asthmatic adults in the high-risk group were more likely to exhibit characteristics associated with asthma, such as decreased lung function, increased blood eosinophil levels, and higher rates of obesity and wheezing than non-asthmatic adults in the average-risk group.

We extensively investigated 41 lifestyle factors reported to be associated with asthma and identified 11 risk factors in the genetically high-risk group for adult-onset asthma. Of these 11 lifestyle factors, eight were obesity-related traits. Obesity is a well-known major risk factor for asthma in children and adults, and one of the hallmarks of obesity is systemic low-grade inflammation with increased levels of many inflammatory markers [40–43]. Thus, inflammation may play a crucial role in the pathogenesis of obesity-related asthma. In particular, both clinical and experimental evidence suggest that leptin, an inflammatory marker, can contribute to the development of asthma by increasing airway hyperresponsiveness [44,45]. High levels of chronic stress are associated with increased interleukin-5 and interleukin-13 levels and eosinophil counts, and these cytokines are believed to play a crucial role in orchestrating airway inflammation and hyperresponsiveness [46,47]. Insomnia and snoring are both risk factors for sleep disorders and have been linked to the development of asthma [48,49]. Recent studies have shown that individuals with insomnia or sleep loss have increased levels of inflammatory markers, such as mononuclear cell nuclear factor κB, and higher levels of high-sensitivity C-reactive protein, which are also associated with asthma-related airway inflammation and obstruction [50–52]. Additionally, snoring is a common symptom of obstructive sleep apnoea syndrome, which is linked to increased oxidative stress and bronchial inflammation due to oxygen desaturation and elevated interleukin-8 levels [53,54]. Obesity, stress, insomnia, and snoring have been identified in previous studies as risk factors for asthma [16,32,55,56]. However, it remains unclear whether the associations of these factors vary depending on genetic risk in adult-onset asthma. Our study demonstrates that, among the 41 candidate factors reported to be associated with asthma, 11 factors related to obesity, stress, insomnia, and snoring are significantly associated with adult-onset asthma in individuals with high genetic risk, similar to those with average genetic risk. Moreover, no significant difference between the high-risk and the average-risk was observed in the effect size of each factor.

Recently, there has been growing evidence that modifying lifestyle factors can reduce the risk of developing diseases such as cardiovascular disease, diabetes, and cancer, even in individuals with a high genetic risk for the disease [39,57–60]. Similar results have been reported for asthma [61]. Using follow-up data from the UKB, Liang et al. emphasised the need for comprehensive interventions, such as modifying lifestyle factors for asthma prevention, even in individuals with a high genetic risk. In this study, we also found that when individuals in the genetically high-risk group maintained a favourable lifestyle, their lifetime risk of asthma was reduced by up to 40% compared to those with an unfavourable lifestyle.

Given the significant burden of primary asthma prevention, there is a critical need for effective preventive strategies, particularly during early life. Early-life interventions can help prevent the onset and mitigate the progression of adult-onset asthma [62]. In particular, the early identification of individuals at high risk for adult-onset asthma due to their unique genetic predispositions could maximise the benefits of such interventions. PRS, which assesses an individual’s genetic susceptibility to specific diseases, are becoming increasingly accurate with the growing sample sizes of GWAS and is expected to play a crucial role in personalised medicine. In this study, we identified individuals at high genetic risk for adult-onset asthma using the recently released standard PRS from the UKB. Subsequently, we evaluated their clinical characteristics and disease onset risk to demonstrate the feasibility of identifying genetically high-risk individuals through PRS. Additionally, we quantified the potential benefits of adjusting lifestyle factors that influence these individuals, thereby suggesting the feasibility of preventive strategies targeting individuals at high genetic risk for adult-onset asthma as candidates for early-life interventions.

While adult-onset asthma is caused by both genetic and lifestyle factors, there is a lack of studies that comprehensively consider the relationship between the two factors. This study investigated the joint association between adult-onset asthma and both genetic and lifestyle factors and confirmed the findings in two independent data sets. However, this study has several limitations. First, as asthma onset was self-reported, the age of asthma onset reported by the participants may have been subject to responder bias. Second, lifestyle factors were evaluated at a single time point during initial recruitment, assuming these behaviours remained unchanged throughout the participants' lives. This assumption could lead to misclassification due to potential changes in lifestyle factors. Additionally, the study design was cross-sectional, hence the association between asthma onset and lifestyle factors cannot be interpreted as causal. Third, the study participants were of white British descent, which may limit the generalisability of the findings to non-European populations. Additional prospective studies involving more diverse populations are needed to validate these findings. Finally, there may be confounding factors that were not accounted for in this study, which could lead to biased results or incorrect interpretations of the observed associations.

## CONCLUSIONS

In conclusion, this study suggests that individuals at a high risk of adult-onset asthma may be identified based on their genetic profiles. Additionally, even individuals with high genetic susceptibility to adult-onset asthma can reduce their lifetime risk by modifying lifestyle factors such as obesity, stress, insomnia, and snoring. Therefore, comprehensive and multifactorial approaches for the prevention of adult-onset asthma should be encouraged to promote behavioural and lifestyle changes in individuals at high genetic risk.

## Additional material


Online Supplementary Document
