# Supplementary material for: Impact of lifestyle factors on adult-onset asthma in genetically high-risk individuals
Source: J Glob Health. 2025 Jun 6;15:04147. doi: 10.7189/jogh.15.04147 (PMC12143115; doi:10.7189/jogh.15.04147)
Supplement: Online Supplementary Document [file jogh-15-04147-s001.pdf]

Supplement to: Kwon SY, Lim JE, Jung H, Baek EJ, Jung Hy, Kang J, Kim T, Oh B. Impact of lifestyle factors on adult-onset asthma in genetically high-risk individuals. J Glob Health. 2025;15:04147.

Figure S1. PRS range of High-risk and Average-risk groups

Figure S2. Cumulative incidence rates of adult-onset asthma according to genetic and lifestyle risk (lifestyle risk score) in the validation dataset

Figure S3. Cumulative incidence rates of adult-onset asthma according to genetic and lifestyle risk (weighted lifestyle risk score) in the validation dataset

Figure S4. Cumulative incidence rates of adult-onset asthma according to genetic risk and each lifestyle factor (Discovery dataset)

Figure S5. Cumulative incidence rates of adult-onset asthma according to genetic risk and each lifestyle factor (Validation dataset)

Table S1. Lifestyle factors associated with asthma

Table S2. Definition of lifestyle status in the UK Biobank

Table S3. Weight for each lifestyle factor in the discovery dataset

Table S4. Basic characteristics of the individuals' adult-onset asthma and controls from the UK Biobank

Table S5. Results of logistic regression analysis in each PRS group

Table S6. Basic characteristics of the high-risk and average-risk groups (Validation dataset)

Table S7. Association analysis between each factor and adult-onset asthma in the high-risk group

Table S8. Association analysis between each factor and adult-onset asthma in the average-risk group

Table S9. Association analysis between each factor and adult-onset asthma in the high-risk and average-risk groups (validation dataset)

Table S10. Distribution of individuals according to lifestyle risk score and each lifestyle factor in the high-risk and average-risk groups

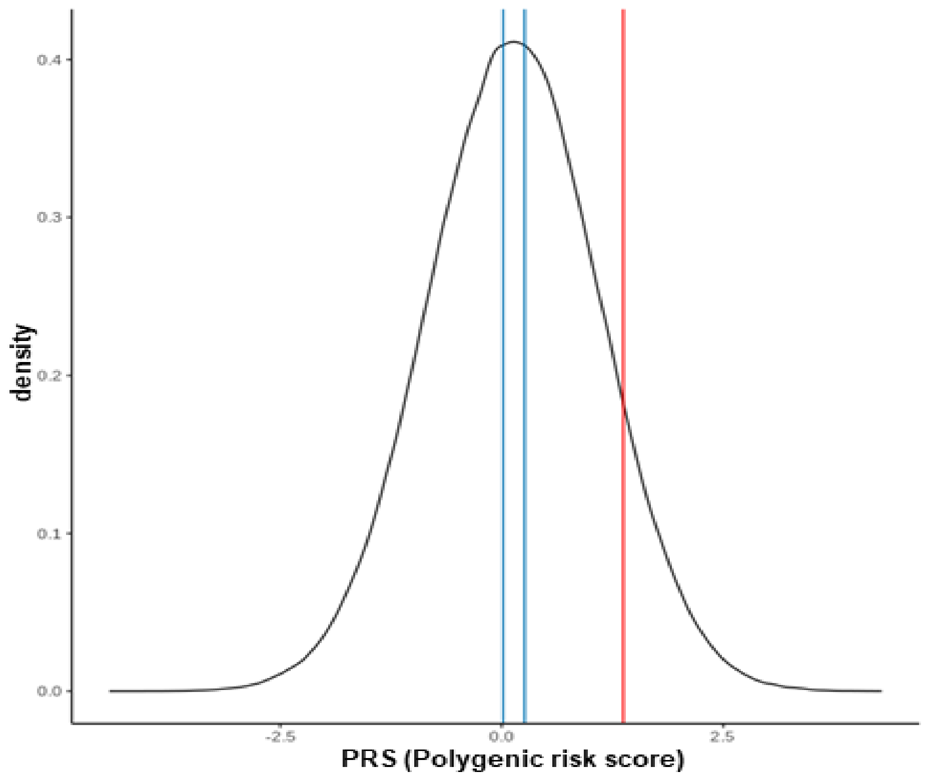

Figure S1. PRS range of High-risk and Average-risk groups.

The PRS range for the high-risk group (PRS > 90%) above the red vertical line is 1.372–4.278, and the PRS range for the average-risk group (PRS 46-55%) between the blue vertical lines is 0.016–0.259.

PRS, polygenic risk score

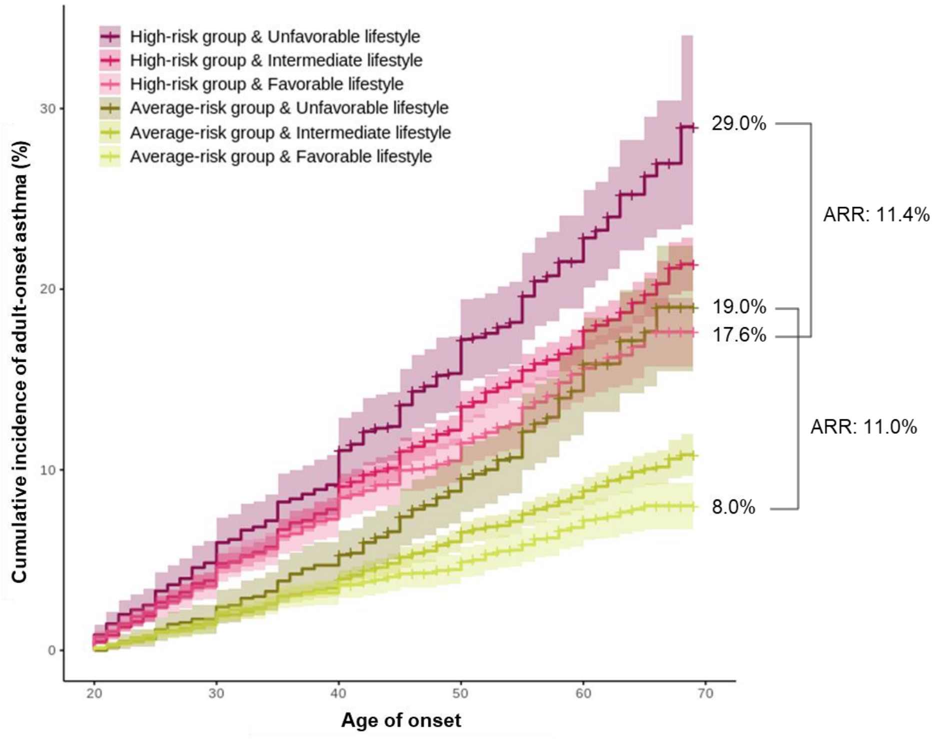

Figure S2. Cumulative incidence rates of adult-onset asthma according to genetic and lifestyle risk (lifestyle risk score) in the validation dataset.

Absolute risk reduction (ARR) is the difference between the lifetime risk of individuals with a favorable lifestyle and those with an unfavorable lifestyle within each genetic risk group. Favorable (score:0), intermediate (score:1-2), and unfavorable (score:3-4) lifestyles were defined based on the lifestyle risk scores.

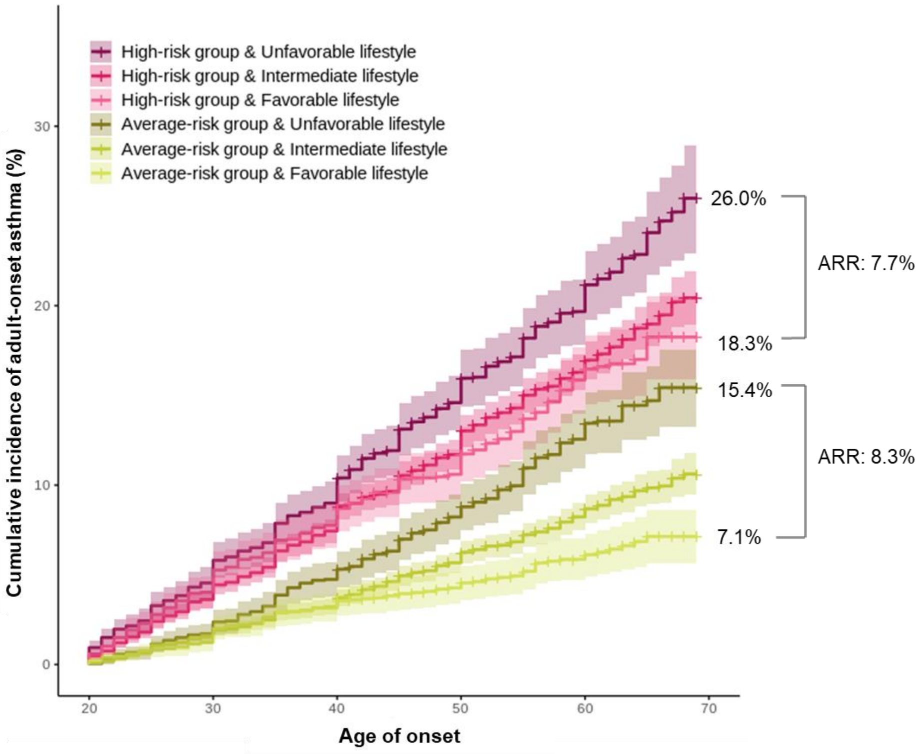

Figure S3. Cumulative incidence rates of adult-onset asthma according to genetic and lifestyle risk (weighted lifestyle risk score) in the validation dataset.

Absolute risk reduction (ARR) is the difference between the lifetime risk of individuals with a favorable lifestyle and those with an unfavorable lifestyle within each genetic risk group. Favorable (lowest quintile), intermediate (quintiles 2–4), and unfavorable (highest quintile) lifestyles were defined based on the weighted lifestyle risk scores.

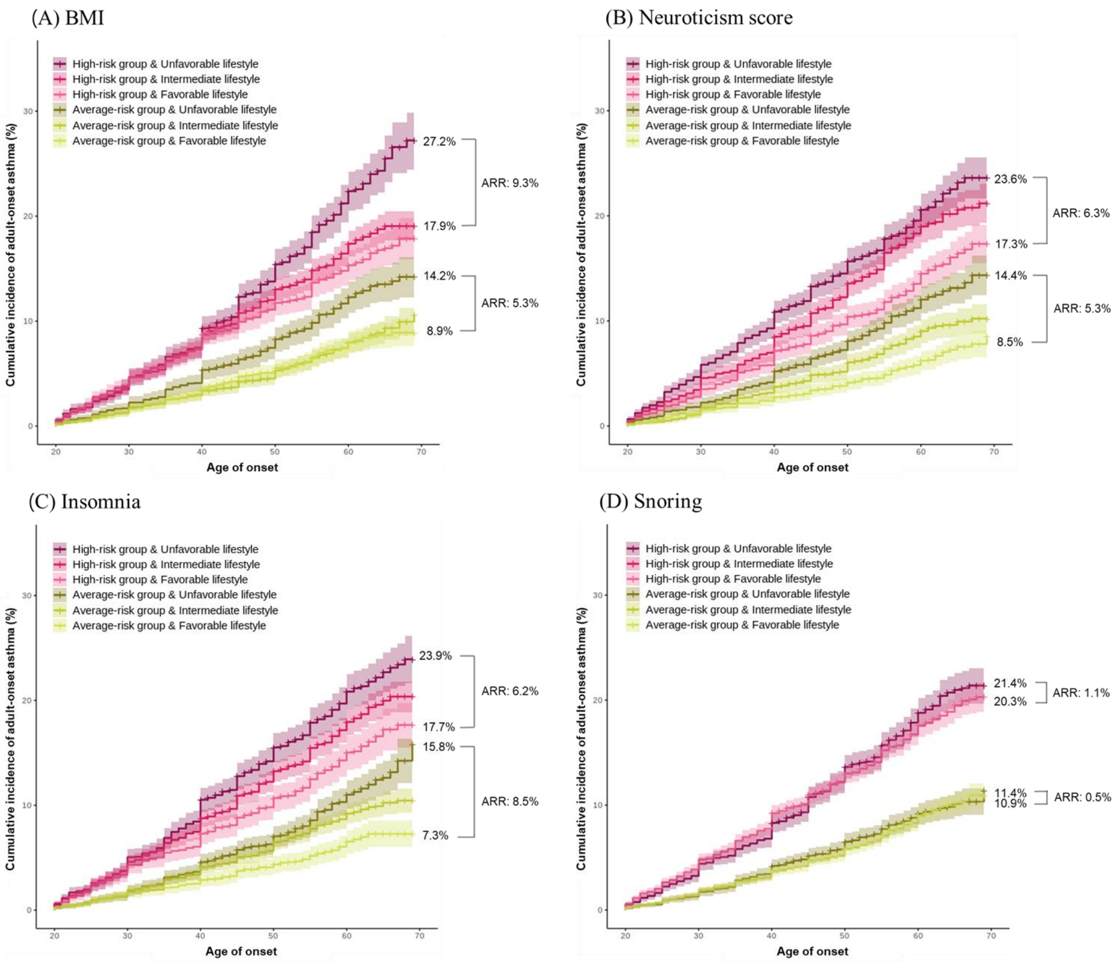

Figure S4. Cumulative incidence rates of adult-onset asthma according to genetic risk and each lifestyle factor (Discovery dataset).

Absolute risk reduction (ARR) is the difference between the lifetime risk of individuals with a favorable lifestyle and those with an unfavorable lifestyle within each genetic risk group.

BMI, body mass index

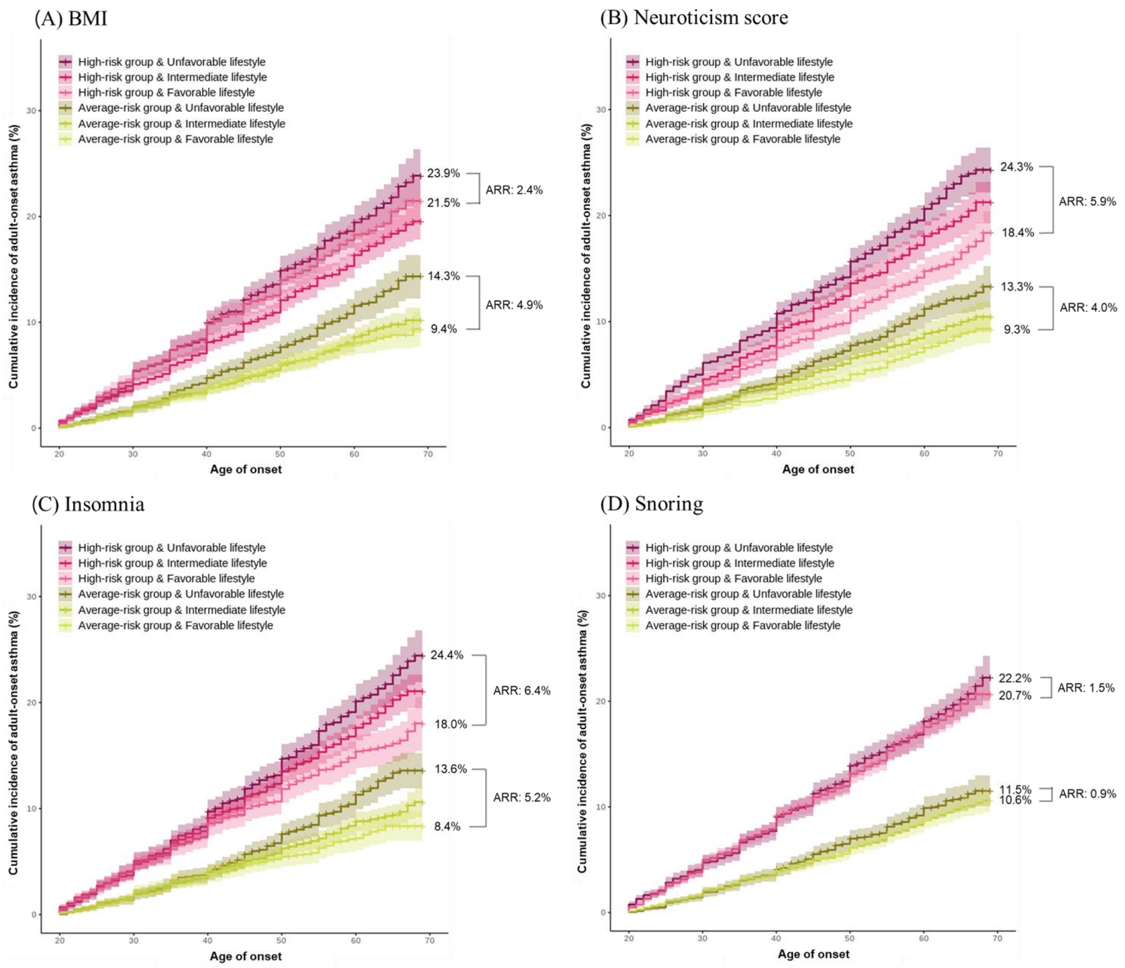

Figure S5. Cumulative incidence rates of adult-onset asthma according to genetic risk and each lifestyle factor (Validation dataset).

Absolute risk reduction (ARR) is the difference between the lifetime risk of individuals with a favorable lifestyle and those with an unfavorable lifestyle within each genetic risk group.

BMI, body mass index

Table S1. Lifestyle factors associated with asthma.

Category	Lifestlye factors	Field	PMID	Year	Title	Jorunal
Diet	Cooked vegetable intake	1289	22628152	2012	Low vegetable intake is associated with allergic asthma and moderate-to-severe airway hyperresponsiveness	Pediatric pulmonolgy
	Salad / raw vegetable intake	1299				
	Fresh fruit intake	1309	28353635	2017	Effects of Fruit and Vegetable Consumption on Risk of Asthma, Wheezing and Immune Responses: A Systematic Review and Meta-Analysis	Nutrients
	Dried fruit intake	1319	28149501	2017	Is fruit and vegetable intake associated with asthma or chronic rhino-sinusitis in European adults? Results from the Global Allergy and Asthma Network of Excellence (GA2LEN) Survey	Clinical and translational allergy
	Oily fish intake*	1329	24265794	2013	Fish and Fish Oil Intake in Relation to Risk of Asthma: A Systematic Review and Meta-Analysis	PLOS ONE
	Processed meat intake*	1349	31147834	2019	Association between processed meat intake and asthma symptoms in the French NutriNet-Santé cohort	European journal of Nutrition
	Cheese intake*	1408	26319606	2015	Diet, interleukin-17, and childhood asthma in Puerto Ricans	Annals of allergy, asthma & immunology
	Salt added to food*	1478	18496466	2008	Wheeze and Asthma in Children: Associations With Body Mass Index, Sports, Television Viewing, and Diet	Epidemology
	Coffee intake	1498	1342314	1992	caffeine intake and asthma symptoms	Annals of epidemiology
Obesity	Body mass index (BMI)	21001	18156567	2008	Body mass index and asthma severity in the National Asthma Survey	Thorax
	Weight	21002	22791994	2012	Asthma and obesity: does weight loss improve asthma control? a systematic review	Journal of Asthma and Allergy
	Waist circumference	48	19706838	2010	Obesity, Waist Size, and Prevalence of Current Asthma in the California Teachers Study Coho	Thorax
	Hip circumference	49				
	Trunk fat mass	23128	30949213	2019	Association between abdominal obesity and asthma: a meta-analysis	Allergy, Asthma & Clinical Immunology
	Trunk fat-free mass	23129				
	Trunk fat percentage	23127				
	Whole body fat mass	23100	24983943	2014	Effects of BMI, Fat Mass, and Lean Mass on Asthma in Childhood: A Mendelian Randomization Study	PLOS Medicine
	Whole body fat-free mass	23101				
	Body fat percentage	23099	23696466	2013	Associations of body fat percent and body mass index with childhood asthma by age and gender	Obesity
Stress	Neuroticism score	20127	19254292	2009	Neuroticism, extraversion, stressful life events and asthma: a cohort study of middle-aged adults	Allergy
Sociodemographic factors	Townsend deprivation index (TDI)	189	12030730	2002	Socioeconomic deprivation and asthma prevalence and severity in young adolescents	European Respiratory Journal
Physical activity	Summed MET minutes per week for all activity	22040				
	Duration of moderate activity	894	23284646	2012	Physical Activity and Asthma: A Systematic Review and Meta-Analysis	PLOS ONE
	Number of days/week of vigorous physical activity 10+ minutes	904				
	Number of days/week of moderate physical activity 10+ minutes	884				
	Duration of walks	874				
	Number of days/week walked 10+ minutes	864				
	Time spent using computer	1080				
	Time spent watching television (TV)	1070	18496466	2008	Wheeze and Asthma in Children: Associations With Body Mass Index, Sports, Television Viewing, and Diet	Epidemology
	Length of mobil phone use*	1110				
	Plays computer games*	2237				
	Weekly usage of mobile phone in last 3 months*	1120				
Local environment	Traffic intensity on the nearest major road	24011	23627489	2013	Ambient wood smoke, traffic pollution and adult asthma prevalence and severity	Respirology
	Close to major road*	24014				
Sun exposure	Time spend outdoors in summer	1050	11844508	2002	Asthma in exercising children exposed to ozone: a cohort study	Lancet
	Time spent outdoors in winter	1060				
	Ease of skin tanning*	1727	21494627	2011	Asthma prevalence associated with geographical latitude and regional insolation in the United States of America and Australia	PLOS ONE
	Use of sun/uv protection*	2267				
Sleep	Sleep duration	1160	32389780	2020	Associations of sleep duration with patient-reported outcomes and health care use in US adults with asthma	Ann Allergy Asthma Immunol
	Insomnia*	1200	34750467	2021	Characterisation of insomnia as an environmental risk factor for asthma via Mendelian randomization and gene environment interaction	Scientific reports
	snoring*	1210	16304284	2005	Association of asthma-related symptoms with snoring and apnea and effect on health-related quality of life	Chest

Field = Code for each lifestyle factor specified by UK Biobank.

*Categorical type factors, the others are continuous or integer types.

Table S2. Definition of lifestyle status in the UK Biobank.

Lifestyle factors	favorable lifestyle	intermediate lifestyle	unfavorable lifestyle	Field ID
BMI[a]	18.5-25 kg/m2	>25 and <30 kg/m2	≥ 30 kg/m2	21001
Neuroticism score[b]	Tertile 1	Tertile 2	Tertile 3	20127
Insomnia	Never/rarely	Sometimes	Usually	1200
Snoring	No	-	Yes	1210

[a]BMI was selected as a representative index of factors related to anthropometry, and lifestyle status was classified according to the criteria provided by the Centers for Disease Control and Prevention (CDC).

[b]The summed score of the 12 questions ranges from 0 to 12 with higher scores indicating higher neuroticism, and lifestyle status was classified by dividing scores into tertiles. Mean score of each status was tertile 1: 0.68 (SD=0.74), tertile 2: 3.50 (SD=0.99), tertile 3: 7.78 (SD=1.95) in the discovery dataset.

BMI, body mass index; SD, standard deviation

Table S3. Weight for each lifestyle factor in the discovery dataset.

Lifestyle factors	beta	se	z	p
BMI[a]	0.267	0.013	20.359	3.8E-92
Neuroticism score[b]	0.196	0.014	14.377	7.2E-47
Insomnia	0.201	0.014	14.440	2.9E-47
Snoring	0.219	0.021	10.291	7.7E-25

[a]BMI was selected as a representative index of factors related to anthropometry, and lifestyle status was classified according to the criteria provided by the Centers for Disease Control and Prevention (CDC).

[b]The summed score of the 12 questions ranges from 0 to 12 with higher scores indicating higher neuroticism, Lifestyle status was classified by dividing scores into tertiles. Mean scores were tertile 1: 0.68 (SD=0.74), tertile 2: 3.50 (SD=0.99), tertile 3: 7.78 (SD=1.95) in the discovery dataset.

se, standard error; BMI, body mass index; SD, standard deviation

Table S4. Basic characteristics of the individuals' adult-onset asthma and controls from the UK Biobank*

Characteristics	Control (n = 238 035)	Case (adult-onset asthma, n = 23 049)
Age in years, x̄ (SD)†	57.06 (7.89)	56.50 (7.99)
Onset age in years, x̄ (SD)	NA	41.67 (12.10)
Onset age in years		
20–29	NA	4140 (17.96)
30–39	NA	5343 (23.18)
40–49	NA	6327 (27.45)
50–59	NA	5309 (23.03)
60–69	NA	1930 (8.37)
Female†	124 880 (52.46)	14 557 (63.16)
Male†	113 155 (47.54)	8492 (36.84)
FEV1/FVC ratio, x̄ (SD)†	77.06 (8.34)	75.09 (9.32)
FEV1% predicted, x̄ (SD)	97.30 (15.82)	89.94 (17.72)
Eosinophil percentage, x̄ (SD)	2.32 (1.42)	2.93 (1.74)
Eosinophil count (10^9 cells/L), x̄ (SD)†	0.16 (0.12)	0.22 (0.17)
BMI in kg/m^2, x̄ (SD)†	27.25 (4.49)	28.18 (5.06)
Obesity†	54 842 (23.17)	7056 (30.94)
Hayfever, allergic rhinitis or eczema†	NA	9404 (40.80)
Wheeze or whistling†	28 983 (12.40)	15 373 (67.75)
Cough on most days†	6329 (10.66)	1603 (28.46)
Bring up sputum on most days†‡	3798 (6.4)	927 (16.46)

BMI – body mass index, FEV1 – forced expiratory volume in one second, FVC – forced vital capacity, NA – not applicable, SD – standard deviation, x̄ – mean

*Presented as n (%) unless specified otherwise. Obesity BMI ≥ 30 kg/m2. Eosinophil percentage is the proportion of eosinophils in the leukocytes.

†Mean or percentage was calculated for individuals with non-missing values. Significant difference between case and control, with $P < 4.2E\text{-}03(0.05/12)$.

Table S5. Results of logistic regression analysis in each PRS group.

	PRS percentile category									
	**PRS < 5 %**	**PRS 5-10%**	**PRS 10-20%**	**PRS 20-40%**	**PRS 40-60% (reference)**	**PRS 60-80%**	**PRS 80-90%**	**PRS 90-95%**	**PRS 95-99%**	**PRS 99-100%**
OR	0.45	0.57	0.64	0.79	-	1.27	1.58	1.98	2.3	3.43
95% CI	0.40-0.50	0.52-0.62	0.60-0.68	0.75-0.83	-	1.22-1.33	1.50-1.66	1.87-2.10	2.17-2.43	3.11-3.78
p-value	3.2.E-50	8.7.E-37	6.5.E-44	3.8.E-23	-	2.7.E-28	1.3.E-74	1.0.E-118	7.2.E-187	2.2.E-137

PRS, polygenic risk score; OR, Odd Ratio; CI, confidence interval

Table S6. Basic characteristics of the high-risk and average-risk groups (validation dataset).

	High-risk group		Average-risk group	
	Control	Case	Control	Case
	N=10,901	*N*=2,149	*N*=11,971	*N*=1,079
Age (years)	57.21 ± 7.79	56.62 ± 7.94	57.09 ± 7.84	56.31 ± 8.12
Onset age (years)[b]	-	40.13 ± 12.33	-	41.59 ± 12.24
onset age 20 - 29 (%)	-	491 (22.85%)	-	200 (18.54%)
onset age 30 - 39 (%)	-	500 (23.27%)	-	255 (23.63%)
onset age 40 - 49 (%)	-	555 (25.83%)	-	286 (26.51%)
onset age 50 - 59 (%)	-	449 (20.89%)	-	244 (22.61%)
onset age 60 - 69 (%)	-	154 (7.17%)	-	94 (8.71%)
Female (%)†	5,672 (52.03%)	1,341 (62.4%)	6,301 (52.64%)	665 (61.63%)
Male (%)†	5,229 (47.97%)	808 (37.6%)	5,670 (47.36%)	414 (38.37%)
FEV1/FVC ratio (%)*†[ab]	76.51 ± 8.5	73.97 ± 9.3	77.13 ± 8.42	75.33 ± 9.14
FEV1% predicted*†[a]	95.62 ± 16.08	88.39 ± 17.55	97.06 ± 15.96	89.84 ± 17.42
Eosinophil percentage (%)*†[ab]	2.59 ± 1.52	3.34 ± 1.84	2.34 ± 1.44	2.94 ± 1.71
Eosinophil count (10^9 cells/Liter)*†[ab]	0.17 ± 0.1	0.22 ± 0.12	0.16 ± 0.1	0.2 ± 0.11
BMI (kg/m^2)*†[a]	27.54 ± 4.62	28.1 ± 5.09	27.28 ± 4.54	28.27 ± 5.17
Obesity (%)*†[a]	2,795 (25.78%)	653 (30.7%)	2,772 (23.27%)	335 (31.54%)
Hayfever, allergic rhinitis or eczema (%)*	-	969 (45.09%)	-	437 (40.5%)
Wheeze or whistling (%)*†[a]	1,621 (15.16%)	1,504 (71.14%)	1,478 (12.6%)	733 (69.48%)
Cough on most days (%)†*	298 (11.22%)	153 (27.77%)	306 (10.22%)	77 (30.08%)
Bring up sputum on most days (%)†*	176 (6.63%)	91 (16.52%)	198 (6.61%)	42 (16.41%)

Data, mean ± standard deviation (SD) or n (%).

Obesity, BMI ≥30 kg/m2; Eosinophil percentage, the proportion of eosinophils in the leukocytes.

[*] Mean or percentage was calculated for individuals with non-missing values.

[†] P < 4.2E-03(0.05/12), comparison of case and control in both groups.

[a] P < 4.2E-03 (0.05/12), comparison of controls between high-risk and average-risk groups.

[b] P < 3.6E-03 (0.05/14), comparison of cases between high-risk and average-risk groups.

FEV1, forced expiratory volume in 1-second; FVC, forced vital capacity; BMI, body mass index.

Table S7. Association analysis between each factor and adult-onset asthma in the high-risk group.

Category	Lifestyle factors	Beta	SE	Z	p^*
Diet	Cooked vegetable intake	0.0360	0.0198	1.82	6.9E-02
	Salad / raw vegetable intake	-0.0086	0.0158	-0.54	5.9E-01
	Fresh fruit intake	-0.0077	0.0174	-0.44	6.6E-01
	Dried fruit intake	-0.0427	0.0281	-1.52	1.3E-01
	Oily fish intake	0.0090	0.0263	0.34	7.3E-01
	Processed meat intake	0.0530	0.0242	2.19	2.9E-02
	Cheese intake	0.0213	0.0229	0.93	3.5E-01
	Salt added to food	0.0577	0.0275	2.10	3.6E-02
	Coffee intake	0.0082	0.0133	0.61	5.4E-01
Obesity	Body mass index (BMI)	0.0422	0.0049	8.63	**6.0E-18**
	Weight	0.0107	0.0016	6.58	**4.7E-11**
	Waist circumference	0.0195	0.0019	10.30	**6.8E-25**
	Hip circumference	0.0173	0.0026	6.75	**1.5E-11**
	Trunk fat mass	0.0376	0.0046	8.16	**3.2E-16**
	Trunk fat-free mass	0.0034	0.0076	0.45	6.5E-01
	Trunk fat percentage	0.0274	0.0034	7.98	**1.4E-15**
	Whole body fat mass	0.0213	0.0026	8.31	**9.9E-17**
	Whole body fat-free mass	0.0064	0.0039	1.66	9.7E-02
	Body fat percentage	0.0342	0.0039	8.83	**1.0E-18**
Stress	Neuroticism score	0.0433	0.0082	5.25	**1.5E-07**
Sociodemographic factors	Townsend deprivation index (TDI)	0.0172	0.0080	2.16	3.1E-02
Physical activity	Summed MET minutes per week for all activity	0.0000	0.0000	-0.85	4.0E-01
	Duration of moderate activity	-0.0004	0.0007	-0.57	5.7E-01
	Number of days/week of vigorous physical activity 10+ minutes	-0.0230	0.0127	-1.81	7.1E-02
	Number of days/week of moderate physical activity 10+ minutes	-0.0240	0.0105	-2.30	2.2E-02
	Duration of walks	-0.0002	0.0007	-0.24	8.1E-01
	Number of days/week walked 10+ minutes	-0.0277	0.0125	-2.22	2.6E-02
	Time spent using computer	0.0432	0.0194	2.22	2.6E-02
	Time spent watching television (TV)	0.0289	0.0160	1.80	7.1E-02
	Length of mobil phone use	0.0358	0.0185	1.94	5.3E-02
	Plays computer games	0.0614	0.0479	1.28	2.0E-01
	Weekly usage of mobile phone in last 3 months	0.0205	0.0209	0.98	3.3E-01
Local environment	Traffic intensity on the nearest major road	0.0000	0.0000	-0.70	4.8E-01
	Close to major road	-0.1283	0.0988	-1.30	1.9E-01
Sun exposure	Time spend outdoors in summer	-0.0121	0.0113	-1.07	2.8E-01
	Time spent outdoors in winter	-0.0234	0.0174	-1.35	1.8E-01
	Ease of skin tanning	0.0137	0.0244	0.56	5.7E-01
	Use of sun/uv protection	-0.0034	0.0273	-0.08	9.0E-01
Sleep	Sleep duration	-0.0334	0.0229	-1.45	1.5E-01
	Insomnia	0.2189	0.0340	6.44	**1.2E-10**
	Snoring	0.2068	0.0516	4.01	**6.1E-05**

* The bold representation indicates a p-value < 1.2E-03 (0.05/41).

SE, standard error

Table S8. Association analysis between each factor and adult-onset asthma in the average-risk group.

Category	Lifestyle factors	Average-risk group (*N*=13,050)		
		beta	se	p*
Obesity	Body mass index (BMI)	0.0497	0.0066	**8.1E-14**
	Weight	0.0143	0.0023	**2.4E-10**
	Waist circumference	0.0205	0.0026	**1.9E-15**
	Hip circumference	0.0190	0.0035	**7.8E-08**
	Trunk fat mass	0.0435	0.0063	**5.5E-12**
	Trunk fat percentage	0.0310	0.0047	**5.5E-11**
	Whole body fat mass	0.0249	0.0035	**1.5E-12**
	Body fat percentage	0.0378	0.0053	**1.2E-12**
Stress	Neuroticism score	0.0563	0.0110	**3.4E-07**
Sleep	Insomnia	0.2509	0.0469	**9.0E-08**
	snoring	0.2099	0.0717	**3.4E-03**

* The bold representation indicates a p-value < 4.5E-03 (0.05/11).
se, standard error

Table S9. Association analysis between each factor and adult-onset asthma in the high-risk and average-risk groups (validation dataset).

Category	Lifestyle factors	High-risk group (*N*=13,050)			Average-risk group (*N*=13,050)		
		beta	se	p*	beta	se	p*
Obesity	Body mass index (BMI)	0.0286	0.0049	**5.2E-09**	0.0474	0.0065	**2.1E-13**
	Weight	0.0086	0.0016	**1.6E-07**	0.0152	0.0021	**1.3E-12**
	Waist circumference	0.0158	0.0019	**5.5E-17**	0.0238	0.0025	**1.1E-21**
	Hip circumference	0.0135	0.0026	**2.0E-07**	0.0204	0.0034	**2.4E-09**
	Trunk fat mass	0.0273	0.0045	**1.4E-09**	0.0463	0.0061	**2.1E-14**
	Trunk fat percentage	0.0215	0.0034	**1.5E-10**	0.0342	0.0046	**1.5E-13**
	Whole body fat mass	0.0149	0.0026	**4.9E-09**	0.0270	0.0033	**7.4E-16**
	Body fat percentage	0.0257	0.0038	**1.3E-11**	0.0424	0.0052	**2.2E-16**
Stress	Neuroticism score	0.0313	0.0081	**1.1E-04**	0.0470	0.0108	**1.4E-05**
Sleep	Insomnia	0.1575	0.0338	**3.1E-06**	0.2987	0.0459	**7.3E-11**
	snoring	0.1942	0.0517	**1.7E-04**	0.1554	0.0697	2.6E-02

* The bold representation indicates a p-value < 2.3E-03 (0.05/22).

SE, standard error

Table S10. Distribution of individuals according to lifestyle risk score and each lifestyle factor in the high-risk and average-risk groups.

Lifestyle factors	High-risk group				Average-risk group			
	favorable lifestyle	intermediate lifestyle	unfavorable lifestyle	Total N^*	favorable lifestyle	intermediate lifestyle	unfavorable lifestyle	Total N^*
**Discovery dataset**								
BMI	3,008 (30.58%)	4,315 (43.87%)	2,513 (25.55%)		3,247 (32.97%)	4,286 (43.53%)	2,314 (23.50%)	
Neuroticism score	3,141 (31.93%)	3,371 (34.27%)	3,324 (33.79%)		3,302 (33.53%)	3,228 (32.78%)	3,317 (33.69%)	
Insomnia	2,479 (25.20%)	4,622 (46.99%)	2,735 (27.81%)	9,836	2,570 (26.10%)	4,680 (47.53%)	2,597 (26.37%)	9,847
Snoring	6,057 (61.58%)	-	3,779 (38.42%)		6,207 (63.03%)	-	3,640 (36.97%)	
Lifestyle risk score[a]	2,461 (25.02%)	6,234 (63.38%)	1,141 (11.60%)		2,670 (27.11%)	6,136 (62.31%)	1,041 (10.57%)	
**Validation dataset**								
BMI	3,067 (30.87%)	4,234 (42.61%)	2,635 (26.52%)		3,264 (32.70%)	4,377 (43.85%)	2,340 (23.44%)	
Neuroticism score	3,313 (33.34%)	3,336 (33.57%)	3,287 (33.08%)		3,341 (33.47%)	3,357 (33.63%)	3,283 (32.89%)	
Insomnia	2,563 (25.80%)	4,679 (47.09%)	2,694 (27.11%)	9,936	2,562 (25.67%)	4,733 (47.42%)	2,686 (26.91%)	9,981
Snoring	6,135 (61.75%)	-	3,801 (38.25%)		6,285 (62.97%)	-	3,696 (37.03%)	
Lifestyle risk score[a]	2,543 (25.59%)	6,237 (62.77%)	1,156 (11.63%)		2,669 (26.74%)	6,272 (62.84%)	1,040 (10.42%)	
Weighted lifestyle risk score[b]	1,861 (18.73%)	5,940 (59.78%)	2,135 (21.49%)		1,940 (19.44%)	6,102 (61.14%)	1,939 (19.43%)	

Data, n (%).

* Participants with information on all four factors in the group.

[a] lifestyle risk scores were calculated as the number of factors with bad lifestyle status, and categorized into three lifestyle categories: favorable (score: 0), intermediate (score: 1-2), and unfavorable (score: 3-4).

[b] Weighted lifestyle risk scores were calculated using weights from the discovery database for each factor, and categorized into three groups, favorable (the lowest quintile), intermediate (quintiles 2–4), and unfavorable (the highest quintile).

BMI, body mass index
